# Biofilm Compositions and Bacterial Diversity on Kitchen Towels in Daily Use

**DOI:** 10.3390/microorganisms13010097

**Published:** 2025-01-06

**Authors:** Yao Zhang, Kexin Li, Yibo Ru, Yue Ma

**Affiliations:** School of Health Science and Engineering, University of Shanghai for Science and Technology, Shanghai 200093, China; 2235053130@st.usst.edu.cn (Y.Z.); 232332332@st.usst.edu.cn (K.L.); 223392694@st.usst.edu.cn (Y.R.)

**Keywords:** biofilm, high-throughput sequencing, bacterial diversity, towel

## Abstract

Towels with complex woven structures are susceptible to biofilm formation during daily use. The composition of biofilms formed on towels used under real-life conditions has yet to be studied. Thus, we investigated the color changes, structural integrity, and biofilm development on towels used continuously for 10 weeks by 12 volunteers in specific kitchen environments. Apparent biofilms composed of bacteria and extracellular polymeric substances (EPSs) were found on all used towels. The bacteria concentrations ranged from 4 to 7 log CFU/g. Proteins were the most abundant EPS, followed by polysaccharides and eDNA. A high-throughput sequencing method was employed to investigate the bacterial diversity on the towels. The predominant bacterial genera differed from towel to towel. *Kocuria*, *Rothia*, *Psychrobacter*, *Enhydrobacter*, and *Pseudomonas* are genera of relatively high abundance that may originate from the human body and foods. In addition, correlations among environmental factors, major bacterial genera, physical properties, and biofilm formation of the towels were analyzed, which could provide a scientific reference for maintaining towel hygiene.

## 1. Introduction

It is common practice for individuals to use towels for personal hygiene maintenance. However, the complex fabric-woven structure of the towels with a high surface area promotes microorganism colonization [1,2]. The microorganisms on towels are diverse and might originate from various sources, such as air, water, human skin, and the surrounding environment [3]. Some microorganisms can adhere, grow, and secrete extracellular substances (EPSs) on the towels, eventually developing into a mature biofilm [4,5]. With the existence of protective EPS matrices, altered metabolic states, and developed quorum sensing systems, microorganisms in the mature biofilm state exhibit high resistance to conventional cleaning methods. These resistance mechanisms manifest through reduced penetration of antimicrobial compounds, lowered antibiotic sensitivity, and the regulation of bacterial community behaviors to adapt to the biofilm environment, contributing to the persistence of biofilms on towels [6,7,8]. Biofilm-contaminated towels are typically associated with reduced hygiene standards and pose a risk of cross-contamination.

The existence of a biofilm causes dullness and reduces the mechanical properties of towels. The deposition of some pigments produced by bacteria might change the color of the towels [9]. For example, pyoverdine and flavin pigments produced by *Pseudomonas aeruginosa* and *Bacillus subtilis* result in yellowish-green and yellowish-brown discoloration, respectively [10,11]. Additionally, the sticky EPS complex may adsorb dust, sweat, and sebum, causing discoloration of the towels [12]. Some bacteria, such as *B. subtilis* and *Caulobacter crescentus*, have been reported to degrade cotton fibers by producing cellulase, resulting in reduced mechanical properties of the cotton fabrics [13,14]. Therefore, understanding the microbial composition of the biofilm on the towels is critical to managing towel usage and maintaining personal hygiene.

The majority of current research related to microorganisms in textiles is more focused on developing functional textiles to control the contamination of microorganisms. For example, N-Halamine-modified polyethylene terephthalate (PET) fabrics containing active chlorine could effectively inactivate typical foodborne pathogens, including Gram-negative *Escherichia coli* and Gram-positive *Listeria innocua* [15]. Cu_2_O nanoparticle-decorated polyethylene fabric inhibited about 98% of *E. coli* biofilm formation [16]. However, in these studies, representative single or multiple microorganisms in planktic or biofilm states were artificially inoculated on the textiles to verify the inhibition performances of the functionalized textiles. It is still uncertain whether artificially inoculated microorganisms and biofilms can reflect real-world conditions and verify the effectiveness of functional textiles. Some studies have attempted to isolate microorganisms from textiles used in real-life conditions. For example, a study found *Salmonella* and *Campylobacter* were widely present on kitchen cloths, yet it did not explore the complete spectrum of microbial communities [17]. Haruto et al. examined bacterial communities and relative abundance on towels used daily via high-throughput sequencing [3]. However, the study did not define specific application scenarios for the tested towels, leading to highly heterogeneous data. Such variability makes it difficult to model textile microbial compositions accurately.

This study investigated, for the first time, the biofilm formed on kitchen towels under real-world conditions, where microorganisms naturally accumulate. This provides a more accurate representation of microbial colonization and biofilm development, offering deeper insights into the hygiene risks of regular household towel use and informing the testing of biofilm-related performance in functionalized textiles. Specifically, 12 volunteers used the towels continuously in a kitchen environment for 10 weeks. After use, the dullness of the towels increased, and the mechanical strength of the warp and weft yarns decreased. SEM images revealed that significant biofilms were formed on the towels, which were composed of microorganisms and their secreted extracellular polymeric substances (EPSs). No yeast or mold was found, and the bacterial concentrations of 12 towels ranged from 4 to 7 log CFU/g. Proteins were the most abundant EPS, followed by polysaccharides and eDNA. In addition, the bacterial communities on different towels were identified through high-throughput sequencing (HTS).

## 2. Materials and Methods

### 2.1. Materials

Cotton towels (25 × 25 cm^2^) were purchased from the local market. Glutaraldehyde solution (25 wt%), ethylenediaminetetraacetic acid (EDTA), Tris-HCl buffer, a Total Carbohydrate Assay Kit, and ethanol were purchased from Sigma-Aldrich (St. Louis, MO, USA). Potato dextrose agar (PDA), rose bengal chloramphenicol (RBC) agar, sodium dodecyl sulfate (SDS), and phosphate-buffered saline (PBS) buffer were purchased from Thermo Fisher Scientific (Waltham, MA, USA). The Bacterial Genomic DNA Rapid Extraction Kit and Pierce^TM^ BCA Protein Assay Kit were purchased from Sangon Biotech (Shanghai, China).

### 2.2. Research Design

**Figure 1:**
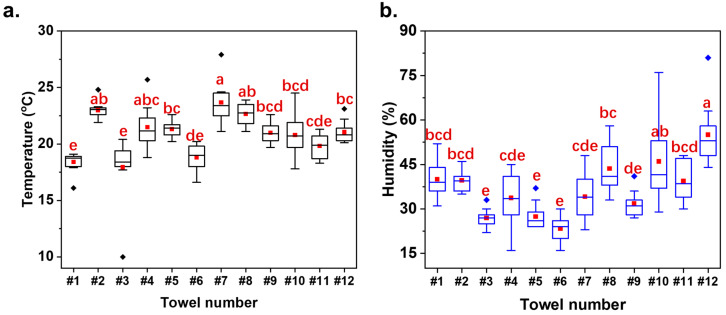
Temperatures (**a**) and humidity (**b**) in the kitchen of 12 volunteers over 10 weeks. The squares and diamonds represent the mean values and outliers, respectively. The letters above the bars indicate the results of group comparisons based on a one-way ANOVA test. The same letters indicate no significant difference (*p* > 0.05), while different letters indicate a significant difference (*p* > 0.05).

12 volunteers were recruited to conduct a ten-week longitudinal test of towels used in a kitchen setting. The users were required to use towels at least 3 times per day for wiping hands after washing, and the towels were hung in or near the kitchen without direct sunlight irradiation. Users were advised to rinse the towel with tap water once per week. In addition, the kitchen temperatures and humidity were collected (Figure 1a,b). This experiment was conducted in the Beijing area from March to May 2023. The average temperatures ranged from 17.94 °C to 23.67 °C. According to the results of Tukey’s multiple comparisons test (*p* < 0.05), it can be categorized into three groups: high temperature (#7, #2, and #8), middle temperature (#10, #9, #12, #5, and #4), and low temperature (#1, #3, #6, and #11). Similarly, the towels were divided into three groups by humidity: high humidity (#8, #10 and #12), middle humidity (#4, #7, #11, #2 and #1), and low humidity (#5, #9, #3, and #6).

### 2.3. Characterizations of Physical Performance

The color change of towels was examined by a reflectance spectrophotometer (Color-eye) and quantitively described by whiteness indexes according to the American Society for Testing and Materials (ASTM) E313 standard [18]. The mechanical performances of wefts, group warps, and pile warps were measured using a universal testing machine Instron 3365 (Norwood, MA, USA) at a constant temperature and humidity (20 ± 2 °C, 65 ± 5%). The elongation speed was fixed at 100 mm min^−1^.

To capture the SEM images of the microorganisms colonizing the towels, the towels were immersed in 2.5% glutaraldehyde overnight at 4 °C to fix the microorganisms. Then, the towels were dehydrated with a series of ethanol/water mixtures with ethanol concentrations of 25%, 50%, 75%, 90%, and 100%. The towel specimens were then coated with gold and made ready for SEM examination.

### 2.4. Measurement of Towel Biofilms

Due to the complex woven structure of the towel, viable microorganisms and EPSs could hardly be isolated. Therefore, the towels were broken into wefts, group warps, and pile warps. These yarns were then cut up and randomly mixed in a 1:1:1 ratio. The viable cell population contaminating the towels was measured based on established standards (GB4789.2-2002; GB4789.15-2016) with mild modification [19,20]. In brief, 2.5 g of yarn mixtures combined with 25 mL of PBS buffer were added into a pre-satirized bag. Subsequently, the sample bags were padded with a homogenizer (Interscience) for 5 min. The homogenized microbial solution was then plated onto PDA and RBC agar to enumerate bacteria and molds, respectively.

The extraction of EPSs from the towels was modified based on the methods described in the literature [4]. The yarn mixture from the used towels was rinsed three times with a 0.85% NaCl solution to remove planktonic cells with biofilm cells left over. A quantity of 1 g of yarn mixtures and 1 mL of buffer solution (pH = 8) containing 10 mM EDTA, 1% SDS, and 10 mM Tris-HCl were placed in a centrifuge tube and vortexed at room temperature for 5 min. Then, the obtained EPS suspension was filtered again using a polyethersulfone membrane (0.22 μm) to remove any residual cells in the biofilm state. The extracted EPS suspensions were stored at −20 °C before testing.

Extracellular DNA (eDNA), protein, and polysaccharide in the extracted EPS suspension were quantified. eDNA was first isolated from the EPS complexes using the Bacterial Genomic DNA Rapid Extraction Kit, and the concentration of eDNA was determined by Nanodrop 2000 (Thermo Scientific) at λ = 260 nm [21]. The concentration of proteins was measured using a Pierce^TM^ BCA Protein Assay Kit, and the polysaccharides were quantified via a Total Carbohydrate Assay Kit according to a published reference [22].

### 2.5. Amoplicon Sequencing of 16S rRNA Genes

The 16S rRNA was amplified using the universal primers 27F: CCGCCTGGGGAGTACG and 1492R: AAGGGTTGCGCTCGTTGC [23]. The PCR amplification conditions were as follows: 94 °C for 5 min followed by 30 cycles of 40 s at 94 °C, 30 s at 55 °C, and 1 min at 72 °C. A final 10 min extension step was done at 72 °C [24]. The PCR amplicons were purified and sequenced by Sangon Biotech Co., Ltd., and the obtained sequences were further analyzed using the BLAST tool from the National Center of Biotechnology Information (NCBI) website.

### 2.6. Statistical Analysis

All experiments were performed in triplicate except for the HTS. The SPSS software package SPSS 20.0 (Chicago, IL, USA) was used for statistical analysis. The data are presented as the mean ± standard deviation. A T-test and a one-way ANOVA were used to assess differences between two and multiple independent groups, respectively. The significance level was set at *p* < 0.05.

## 3. Results and Discussions

### 3.1. Physical Performance

**Figure 2:**
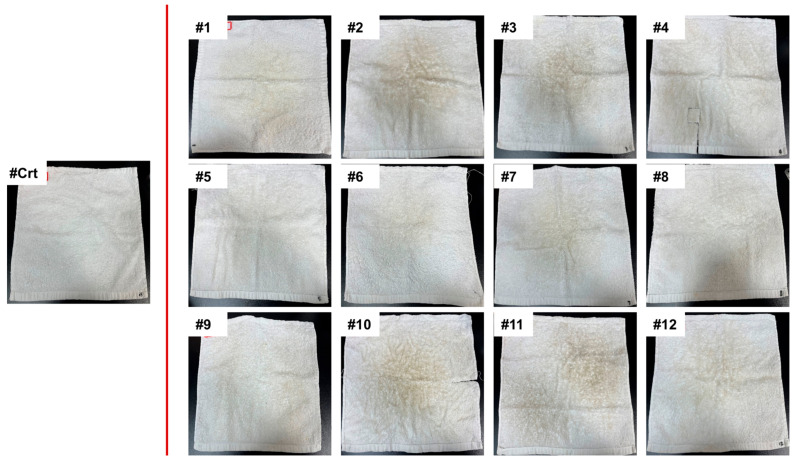
Optical images of new towel (control) and 10-week used towels.

**Figure 3:**
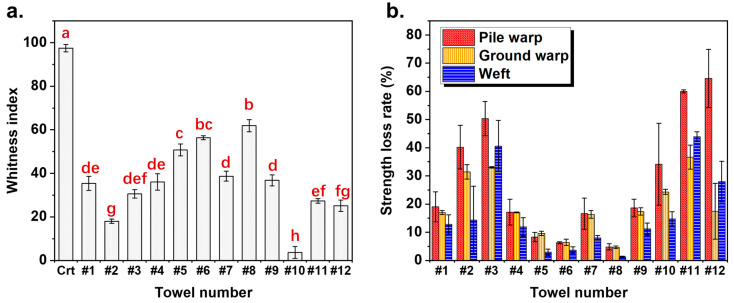
(**a**) Whiteness index of the new towel and 10-week used towels (The letters above the bars indicate the results of group comparisons based on a one-way ANOVA test. The same letters indicate no significant difference (*p* > 0.05), while different letters indicate a significant difference (*p* > 0.05). (**b**) Strength loss rates of pile warp, ground warp, and weft of 10-week used towels compared to those of the new towel.

As shown in Figure 2, all used towels turned yellow, a change which could be detected by the naked eye. A reflectance spectrophotometer was employed to evaluate the color change of the towels quantitatively. The average whiteness index (WI) of the control towels was 97.72, indicating a near-standard white color. However, after being used for ten weeks, the WI values of all towels decreased significantly (Figure 3a, *p* < 0.05). Towel #10 demonstrated the lowest WI of 3.69, implying the highest level of dullness, which was consistent with the naked-eye observation results. In addition, towels #5, #6, and #8 displayed high WI values, indicating relatively low dullness levels.

A new towel demonstrated promising mechanical properties with pile warp-, ground warp-, and weft yarn-breaking strengths of 2.71, 8.89, and 4.75 N, respectively. As shown in Figure 3b, the fibers and yarns might fatigue and break during the application period due to repeated friction and stretching, reducing the overall strength of all the tested towels. For example, towel #11 showed the most significant reduction in breaking strengths, with the pile warp, ground warp, and weft yarns decreasing by 60.0%, 36.7%, and 43.9%, respectively. For all used towels, the pile warp yarns on the towel surface demonstrated a higher strength loss rate than the ground warp and weft yarns, possibly because the pile warp yarns were subjected to higher pressures and tensile forces. Generally, towels with higher whiteness exhibited less loss of mechanical strength. This phenomenon may be attributed to the adherence of sebum, which facilitates the colonization of microorganisms [25]. Consequently, an increased secretion of metabolites by these microorganisms may adhere to the fabric surfaces, resulting in increased surface roughness and accelerated fabric abrasion.

### 3.2. Biofilm Formation

**Figure 4:**
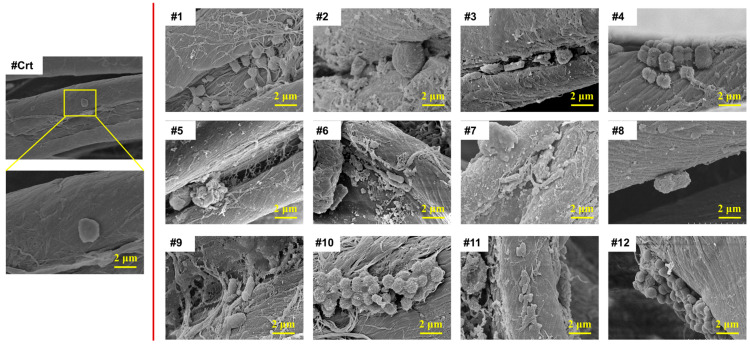
SEM images of new towel (control) and 10-week used towels.

The morphology of towels was observed via SEM imaging. As shown in Figure 4, new towels showed little evidence of bacterial colonization, except for occasional small patches of individual bacterial cells without EPSs. In sharp contrast, all 12 used towels displayed significantly greater densities of microorganisms than the control towel. Various microcolony morphologies could be found on the same piece of towel, suggesting the presence of different microorganism species. Most of the microorganisms were located in the gaps between cotton fibers and grooves of kidney-shaped cotton fibers, which were difficult to rinse off with water. In addition, all used towels demonstrated the presence of EPSs around the microorganisms, indicating biofilm development. Microbial adhesion to the towels seems to be associated with the confirmed color changes. For example, minimal microbial adhesion and EPS-like substances were found on towel #8, which displayed the whitest color with a WI of 62.91. However, for towel #10, considered the dirtiest towel with the smallest WI of 3.69, a large number of adhered microorganisms appeared not only in the gaps between cotton fibers but also on the relatively smooth fiber surface. These SEM results confirmed that microbial colonization and further production of EPSs are important indicators for the assessment of towel cleanliness during daily usage.

**Figure 5:**
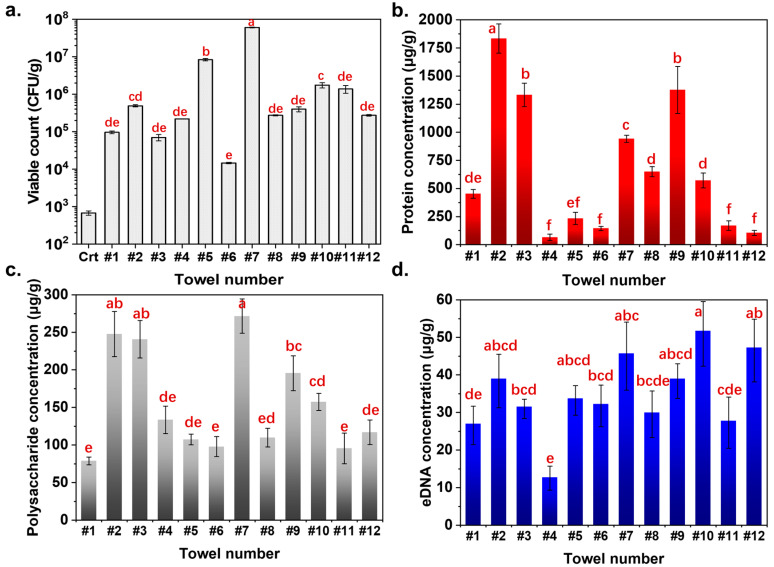
(**a**) Bacterial population of control and 10-week used towels. (**b**) Protein concentrations, (**c**) polysaccharide concentrations, and (**d**) eDNA concentrations in the biofilm of 10-week used towels. The letters above the bars indicate the results of group comparisons based on a one-way ANOVA test. The same letters indicate no significant difference (*p* > 0.05), while different letters indicate a significant difference (*p* > 0.05).

The viable cells contaminated on the used towels were estimated via a standard plate counting method. As shown in Figure 5a, about 6.75 × 10^2^ CFU/g bacteria were detected on the unused towel. After ten weeks of usage, the bacterial population on the towel significantly increased by nearly 10^2^~10^5^ times. Towels #6 and #7 demonstrated the lowest and the highest contamination levels with bacterial concentrations of 1.46 × 10^4^ and 6.1 × 10^7^ CFU/g, respectively. The bacterial concentrations on most of the towels were about 10^5^ CFU/g. However, no fungus was found on any of the 12 towels.

Besides viable cell concentration, the other important biofilm components are EPSs, including polysaccharides, proteins, and eDNA. Considering the low bacterial load and the absence of adhesive structure (Figure 4) of the control towels, it was concluded that no biofilm was formed on the control towels. Consequently, EPS component analysis was not performed on the control towels. As illustrated in Figure 5b–d, proteins are the most predominant EPS component, followed by polysaccharides and eDNA, except for towel #12. Protein concentration varies significantly among towels, with towel #2 having the highest concentration (1832.84 μg/g), 28 times higher than towel #4 (65.26 μg/g). The content of polysaccharides and eDNA on different towels ranged from 78.81~271.67 μg/g and 12.75~51.75 μg/g, respectively, which did not vary as much as proteins. The principal source of variation in the EPS components was the differential production of proteins by microorganisms. Theoretically, the higher the concentration of microorganisms on the towel, the more EPSs it contains. For example, only 97.86 μg/g, 144.48 μg/g, and 32.35 μg/g of polysaccharides, proteins, and eDNA could be found on towel #6 with a low bacterial contamination level of 1.46 × 10^4^ CFU/g. More viable cells (6.1 × 10^7^ CFU/g) colonizing towel #7 secreted more EPSs with polysaccharide, protein, and eDNA concentrations of 271.67 μg/g, 941.62 μg/g, and 45.75 μg/g, respectively. However, the EPS contents in the towel are not always positively correlated with the number of viable cells colonizing it. For example, only about 4 log CFU/g of bacteria were found to be colonizing towel #2, while its overall EPS contents were almost the same as that of towel #7, which contained 7 log CFU/g of viable bacteria. Such phenomena might be attributed to the different types of microbes colonizing different towels, leading to variations in EPS contents. Therefore, further analysis of the microbiome is needed.

### 3.3. Microbiome Analysis of Towel Biofilms

After quality filtering, 716,160 valid sequence reads were generated based on 24 sample sequencing results (control towels with limited levels of microorganisms were unable to generate sufficient sequence data). Two different locations were selected for testing on each towel, and the control group could not produce a valid result due to the limited microorganism population. As shown in Table 1, a total of 2256 amplicon sequence variants (ASVs) were identified following the clustering of all samples (ranging from 26 to 405). HTS exhibited good sequencing coverage with all coverage indexes >99.9%. The observed ASV, Chao1, Shannon, and Simpson indicated the microbial diversity in the samples. A negative correlation was observed between the bacterial contamination level and the microbial diversity presented. For example, towel #6, with the lowest bacterial contamination level, displayed the highest ASV, chao1, and Shannon indexes and the lowest Simpson index, indicating the highest species richness. This phenomenon might be attributed to the fact that when the bacterial population is high, the dominant strains inhibit the growth of other strains, resulting in a decrease in microbial diversity [26].

**Figure 6:**
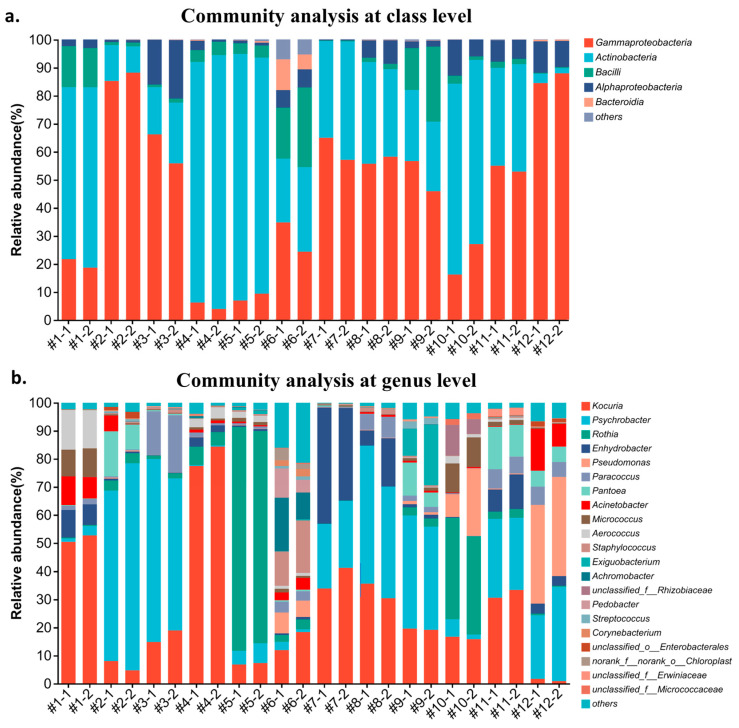
Bacterial community bar plot analysis of 10-week used towels at (**a**) class and (**b**) genus levels.

Figure 6a,b display the complexity of the bacterial community on towels at class and genus levels, respectively. The bar plots show the relative abundance of each class or genus, with different colors representing bacterial groups and the bar height indicating their abundance in each sample. The disparity in the microbial composition between the two parallel samples is minimal, proving the reliability of the HTS result. Actinobacteria was the most dominant class on towels #1, #4, #5, and #10. However, the dominant class was identified as Gamma-proteobacteria for towels #2, #3, #7, #8, #9, #11, and #12. Specifically, towel #6, with the lowest bacterial contamination level, contained three major bacterial classes (Actinobacteria: 29.49%, Gamma-proteobacteria: 26.46%, and Bacilli: 23.30%), displaying the richest bacterial diversity at the class level. Another study of microbial populations on daily towels found Gamma-proteobacteria, Actinobacteria, and Alpha-proteobacteria to be the predominant bacterial classes, but no Bacilli could be detected [3]. This difference suggests that the high relative abundance of Bacilli might be related to the kitchen environment in which the towels were used in this study. Several studies confirm this hypothesis. One study identified 113 *Bacillus* isolates from kitchen roll samples, while another study detected *Bacillus* spp. in food residues from kitchens [27,28]. *Bacillus* species are commonly introduced into kitchens through protein- or starch-rich foods. Their spores are highly heat-resistant, enabling them to survive high temperatures and even endure some cooking processes. Moreover, the warm, humid conditions in kitchens promote the germination and growth of these spores, making it likely to detect *Bacillus* on kitchen towels.

At the genus level, the relative abundance of *Kocuria*, belonging to the Actinobacteria class, on towels #1, #4, #7, #8, and #11 was significant. *Kocuria* usually inhabits humans skin and mucous membranes [29]. However, another study isolated *Kocuria* from both kitchen floors and aprons, suggesting that *Kocuria* might be present in the kitchen environment [30]. The major genus contaminating towels #5 and #10 was *Rothia* (Actinobacteria), which is a common bacterium of the oral cavity [31]. A study detected *Rothia* in both the indoor air and sinks in kitchen environments. Therefore, *Rothia* is likely transmitted to the towels through aerosols or direct contact cross-contamination [32]. The dominant genus in towels #2, #3, #8, #9, and #11 was *Psychrobacter*, belonging to the gamma-proteobacteria class, which might initially come from chilled proteinaceous foods, such as fish, poultry, and dairy products [33,34]. *Enhydrobacter* and *Pseudomonas*, belonging to the gamma-proteobacteria class, were the dominant genera on towels #7 and #12, respectively. There were few reports on the former genera. The only known species is *Enhydrobacter aerosaccus*, isolated from Wintergreen Lake, Michigan [35]. The *Enhydrobacter* identified from towel #7 showed 97% similarity to *Enhydrobacter aerosaccus*. However, the specific species must be tested for physiological and biochemical characteristics for further determination. In addition, *Pseudomonas* is an important spoilage organism in foods, and its high abundance on towel #12 may be due to cross-contamination caused by the contact of the user with the spoiled foods [36].

Overall, the microbial diversity observed on different towels can be attributed to the distinct human and diverse environmental microbiota. Staphylococci are abundant bacteria in the human skin microbiome [37]. Some species, particularly *Staphylococcus aureus* and *Staphylococcus epidermidis*, are opportunistic pathogens and cause significant diseases [38]. Interestingly, *Staphylococcus*, with a relative abundance of 14.94%, was detected on only towel #6, which had the lowest bacterial concentration. Such a phenomenon indicated that staphylococcal colonization in towels was not advantageous and would disappear as the overall bacterial concentration increased. Contrary to common sense, skin infections induced by opportunistic pathogenic *Staphylococcus* are more likely to occur with relatively clean towels. Besides *Staphylococcus,* the dominant bacterial genera on towels also contain strains of opportunistic pathogenic bacteria. For example, the infection of *Kocuria kristinae*, an opportunistic pathogen, might cause catheter-related bacteremia and infective endocarditis when the human skin is broken or the immune system is compromised [39]. If *Kocuria kristinae* accidentally enters the bloodstream, it can lead to sepsis, which may manifest as symptoms such as fever, chills, low blood pressure, and multi-organ failure. Therefore, the use of clean towels is necessary to maintain personal hygiene, especially in environments where vulnerable individuals may be exposed.

### 3.4. Correlation Discussion

**Figure 7:**
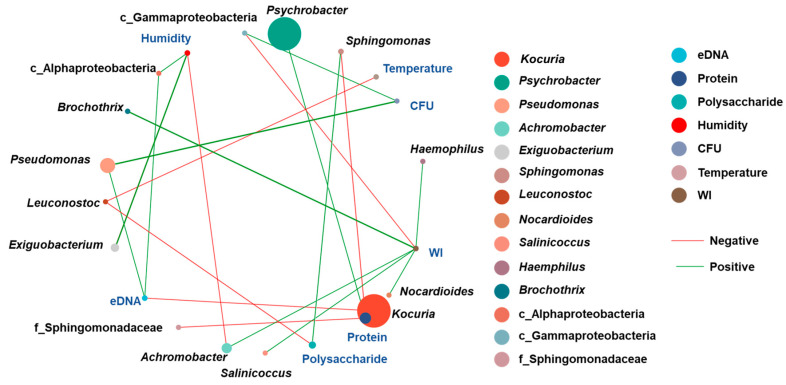
Network analysis of bacterial genus, environmental factors, whiteness index, and biofilm components. Red and green lines indicate negative and positive correlations, respectively.

Spearman’s rank correlation tests were employed to analyze the correlations among the environmental factors and constituent bacterial genera. As shown in Figure 7, factors that were significantly correlated (*p* < 0.05) and had highly significant correlation coefficients (|ρ| > 0.4) were connected by a line. Red and green lines indicate the negative and positive correlations, respectively.

There was no direct correlation among the environmental factors, including temperature, humidity, whiteness index (WI), total plate counting (CFU), and EPSs. However, the correlations between environmental factors and various bacterial genera could be identified. The experiments were conducted from March to May 2023 in Beijing. The temperature variations were not significant (Figure 1a). Only *Leuconostoc*, a typical psychrophilic bacterial genus, demonstrated a negative correlation with the temperature [40]. Theoretically, increasing ambient humidity could increase water availability for microbial growth. However, high humidity might also lead to insufficient oxygen supply, inhibiting some aerobic species of *Achromobacter* [41]. Thereby, a negative correlation was found between humidity and *Achromobacter*, which usually occurs in water and soils [42]. The WI of the towel was positively correlated to *Brochothrix*, *Haemophilus*, *Nocardiodies*, and *Achromobacter*, and negatively correlated to unclassified gamma-proteobacteria. Moreover, the more unclassified gamma-proteobacteria and *Pseudomonas* existed, the higher the bacterial contamination level achieved.

*Psychrobacter* was positively correlated with the production of extracellular proteins. A study reported that *Psychrobacter* produces cold attachment protein 1 (Cat1), accelerating biofilm formation [43]. This led to the hypothesis that the presence of *Psychrobacter* plays an essential role in forming robust biofilms in towel environments. eDNA was positively related to unclassified alpha-proteobacteria. However, a negative correlation was observed between eDNA and *Kocuria*, which was the predominant genus colonizing towels #1, #4, #7, #8, and #11. Research also found that *Kocuria calsicia* isolated from a meat-processing environment could not produce eDNA due to the lack of biofilm-associated genes, which was consistent with the findings of this work [44]. In addition, *Sphingomonas* distributed in nature and tap water showed a positive correlation with polysaccharides, which agrees with previous reports. It was found that *Sphingomonas* exhibited a great ability to produce exopolysaccharides, which aid in irreversible attachment and biofilm formation [45,46].

## 4. Conclusions

The study systematically analyzed 12 towels used in kitchen environments for 10 weeks. All towels exhibited evident biofilm formation, which resulted in increased dullness and decreased mechanical performance. No yeast or mold was detected, but bacterial colonization ranged from 4 to 7 log CFU g^−1^. *Kocuria*, *Rothia*, *Psychrobacter*, *Enhydrobacter*, and *Pseudomonas* displayed high relative abundance. However, the predominant bacterial genera varied among towels. In addition, quantitative analysis of EPSs in biofilm indicated that proteins were the most abundant, followed by polysaccharides and extracellular DNA (eDNA). Based on these findings, it is recommended to wash kitchen towels regularly with hot water and detergent to reduce bacterial growth and biofilm buildup. Towels exhibiting visible dullness or reduced mechanical performance likely indicate biofilm development and should be replaced or disinfected promptly. In sum, these results provide a foundation for developing a reliable biofilm model on towels, which can inform improvements in household hygiene practices and contribute to better maintenance and management of kitchen textiles.

## Figures and Tables

**Table 1:** ASV, Chao1, Shannon, Simpson, and coverage of HTS results.

Towel		ASV	Chao1	Shannon	Simpson	Coverage
#1	#1-1	50.00	76.75	1.89	0.25	>99.9%
	#1-2	43.00	66.00	1.90	0.26	>99.9%
#2	#2-1	74.00	124.79	1.75	0.38	>99.9%
	#2-2	82.00	133.65	1.45	0.54	>99.9%
#3	#3-1	54.00	99.00	1.80	0.26	>99.9%
	#3-2	67.00	104.27	2.02	0.21	>99.9%
#4	#4-1	61.00	102.50	1.31	0.56	>99.9%
	#4-2	42.00	65.46	0.93	0.67	>99.9%
#5	#5-1	59.00	95.00	1.14	0.63	>99.9%
	#5-2	74.00	155.05	1.35	0.57	>99.9%
#6	#6-1	298.00	348.17	3.91	0.06	>99.9%
	#6-2	405.00	558.73	4.41	0.04	>99.9%
#7	#7-1	30.00	41.17	1.56	0.26	>99.9%
	#7-2	26.00	43.43	1.59	0.24	>99.9%
#8	#8-1	60.00	101.59	2.14	0.18	>99.9%
	#8-2	72.00	109.38	2.21	0.17	>99.9%
#9	#9-1	111.00	199.94	2.93	0.11	>99.9%
	#9-2	101.00	153.42	2.82	0.12	>99.9%
#10	#10-1	67.00	104.27	2.40	0.17	>99.9%
	#10-2	63.00	91.50	2.17	0.19	>99.9%
#11	#11-1	85.00	159.09	2.76	0.13	>99.9%
	#11-2	85.00	133.35	2.71	0.13	>99.9%
#12	#12-1	130.00	217.97	2.85	0.17	>99.9%
	#12-2	117.00	198.20	2.40	0.23	>99.9%

## Data Availability

The original contributions presented in this study are included in the article. Further inquiries can be directed to the corresponding author.
